# Cathepsin L Inhibition Prevents Murine Autoimmune Diabetes via Suppression of CD8^+^ T Cell Activity

**DOI:** 10.1371/journal.pone.0012894

**Published:** 2010-09-22

**Authors:** Akiko Yamada, Naozumi Ishimaru, Rieko Arakaki, Nobuhiko Katunuma, Yoshio Hayashi

**Affiliations:** 1 Department of Oral Molecular Pathology, Institute of Health Biosciences, The University of Tokushima Graduate School, Tokushima, Japan; 2 Institute of Health Science, Tokushima Bunri University, Tokushima, Japan; Universität Würzburg, Germany

## Abstract

**Background:**

Type 1 diabetes (T1D) is an autoimmune disease resulting from defects in central and peripheral tolerance and characterized by T cell-mediated destruction of islet β cells. To determine whether specific lysosomal proteases might influence the outcome of a T cell–mediated autoimmune response, we examined the functional significance of cathepsin inhibition on autoimmune T1D-prone non-obese diabetic (NOD) mice.

**Methods and Findings:**

Here it was found that specific inhibition of cathepsin L affords strong protection from cyclophosphamide (CY)-induced insulitis and diabetes of NOD mice at the advanced stage of CD8^+^ T cell infiltration via inhibiting granzyme activity. It was discovered that cathepsin L inhibition prevents cytotoxic activity of CD8^+^ T cells in the pancreatic islets through controlling dipeptidyl peptidase I activity. Moreover, the gene targeting for cathepsin L with application of *in vivo* siRNA administration successfully prevented CY-induced diabetes of NOD mice. Finally, cathepsin L mRNA expression of peripheral CD8^+^ T cells from NOD mice developing spontaneous T1D was significantly increased compared with that from control mice.

**Conclusions:**

Our results identified a novel function of cathepsin L as an enzyme whose activity is essential for the progression of CD8^+^ T cell-mediated autoimmune diabetes, and inhibition of cathepsin L as a powerful therapeutic strategy for autoimmune diabetes.

## Introduction

The cathepsins constitute a family of lysosomal proteases that are recognized as non-specific scavengers to recycle cellular proteins within lysosomes, and that are also found to display cell type–specific functions [1]–[5]. Although the action of lysosomal proteases is not necessarily limited to the endosomal system for antigen processing, it remains obscure in that the *in vivo* activity of lysosomal proteases regulates the peripheral immune responses.

Studies of gene-knockout mice or specific inhibitors for cathepsins have demonstrated that the emzymatic activities of cathepsins play key roles in the pathogensesis of autoimmune diseases such as autoimmune myasthenia gravis, rheumatoid arthritis, Sjogren's syndrome, and autoimmune Type-1 diabetes (T1D) [6]–[9]. As it was reported that cathepsin L-deficient NOD mice are protected from insulitis and diabetes due to the increased number of regulatory T (Treg) cells in the periphery through the defective thymic selection [10], the therapeutic application with cathepsin inhibitor may be clinically useful. However, it is still unclear whether cathepsin L plays any role in peripheral effector cells or cytotoxic cells other than Treg cells in the development of autoimmune diabetes.

Here the role of cathepsin L in cyclophosphamide (CY)-treated nonobese diabetic (NOD) mice, a model for spontaneous T1D [11], [12] using a cathepsin L-specific inhibitor was investigated. It has been described that CY-induced T1D in the NOD mouse is associated with a reduction of Treg cells [13]. In cathepsin L-deficient mice and cathepsin L-deficient NOD mice, the direct effect of cathepsin L on the peripheral T cells has not been clarified; although it was demonstrated that cathepsin L plays a key role in the T cell differentiation in the thymus [10], [13].

In this study, a novel function of cathepsin L as an enzyme whose activity is essential for the progression of CD8^+^ T cell-mediated autoimmune diabetes was identified, and the specific inhibition of cathepsin L as a powerful therapeutic strategy for autoimmune diabetes was demonstrated.

## Results

### Effective Treatment of CY-induced T1D in NOD Mice by a specific inhibitor of cathepsin L

**Figure 1:**
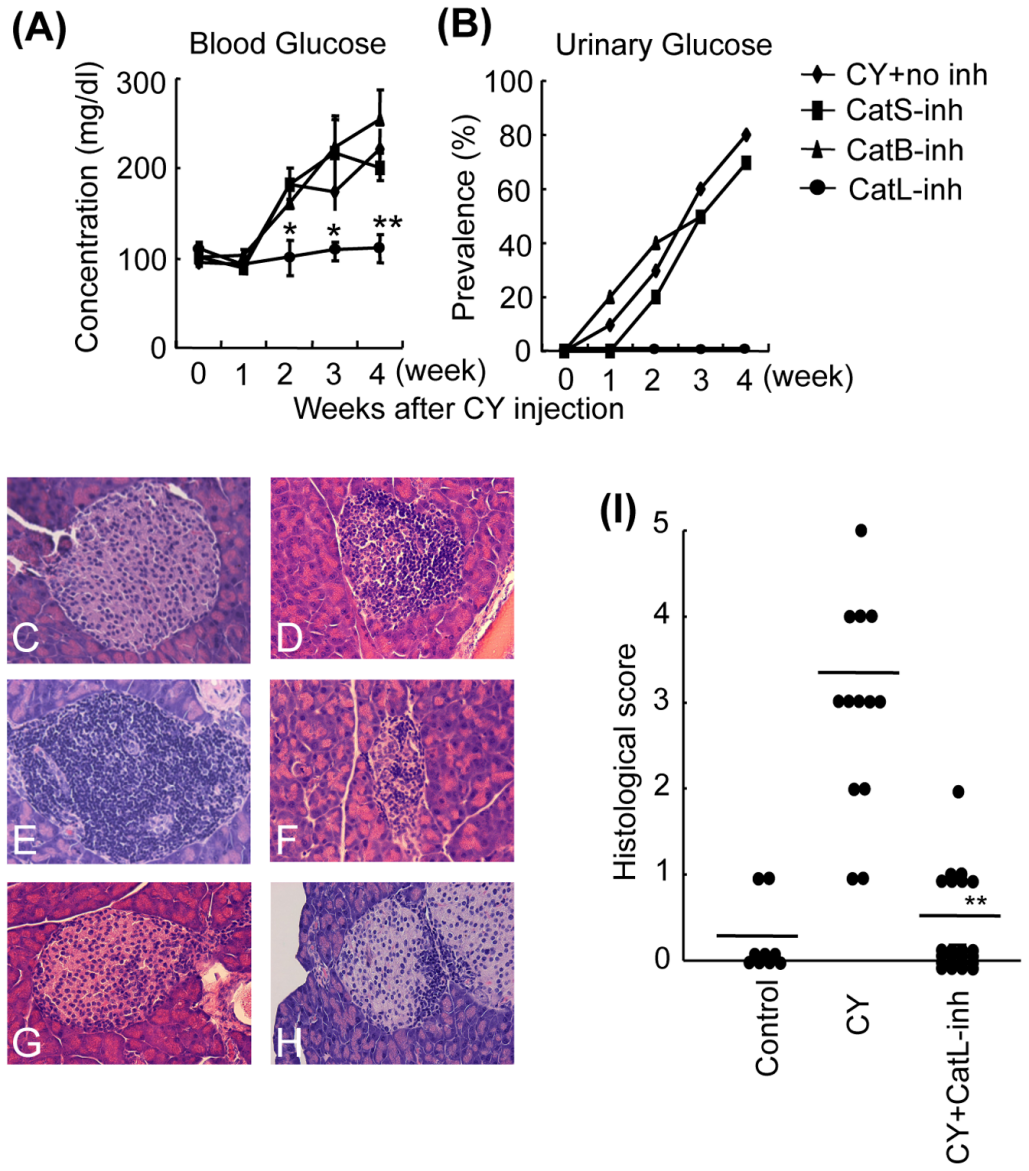
*In Vivo* Therapeutic Effect of Cathepsin L Inhibitor (CatL-inh) on T1D in CY-treated NOD Mice. (A, B) The blood glucose and urine glucose levels were monitored weekly. Data are shown as means ± s.d. of 4 or 5 mice in each group. *, *P*<0.05, and **, *P*<0.01. CY-treated mice versus CatL-inh-treated mice. Results are representative of 4 or 5 mice in each group. (C, D, E, F, G, H) Histopathological analysis of islets control (prediabetic) (C), CY-treated (D, E, F) and CY+CatL-inh-treated (G, H) NOD mice. Results are representative of 4 or 5 mice in each group. (I) Histological score of islets from control (n = 5), CY-treated (n = 4) and CY+CatL-inh-treated (n = 5) NOD mice. Data are shown as histological score of individual islets.

A specific inhibitor of the cathepsin L (CatL-inh) [14], [15] was intraperitoneally (i.p.) administered into CY-treated NOD mice. Treatment with CatL-inh protected diabetes-prone NOD mice from subsequently occurring diabetes such as high blood sugar and urine sugar while there was no therapeutic effect of cathepsin B inhibitor (CatB-inh) and cathepsin S inhibitor (CatS-inh) (Figure 1A, B). To determine whether CatL-inh administration is effective on the onset of insulitis, histological sections for the presence of insulitis were analyzed. The histological finding of islets from CY-treated NOD mice showed lymphocytic infiltration, ranging from peri-insulitis to severe extensive, and atrophic or damaged islets with a decrease in cell numbers (Figure 1D, E, F), compared with that of control NOD mice (Figure 1C). In contrast, CatL-inh protected severe insulitis and damage of islets, although a slight inflammatory lesion with peri-insulitis was observed (Figure 1G, H). Moreover, semiquantitative evaluation of islet inflammation was performed using pancreatic sections from control, CY-treated, and CY+CatL inh-treated NOD mice. The histological score of CY-treated NOD mice was significantly reduced by the administration of CatL inh (Figure 1I). This result showed that cathepsin L plays an important role in the onset of T1D in NOD mice as previously described [10], and that the specific cathepsin L inhibitor may be useful for the effective treatment of autoimmune diabetes.

### Effects of CatL-inh Administration on Peripheral Immune Cells

**Figure 2:**
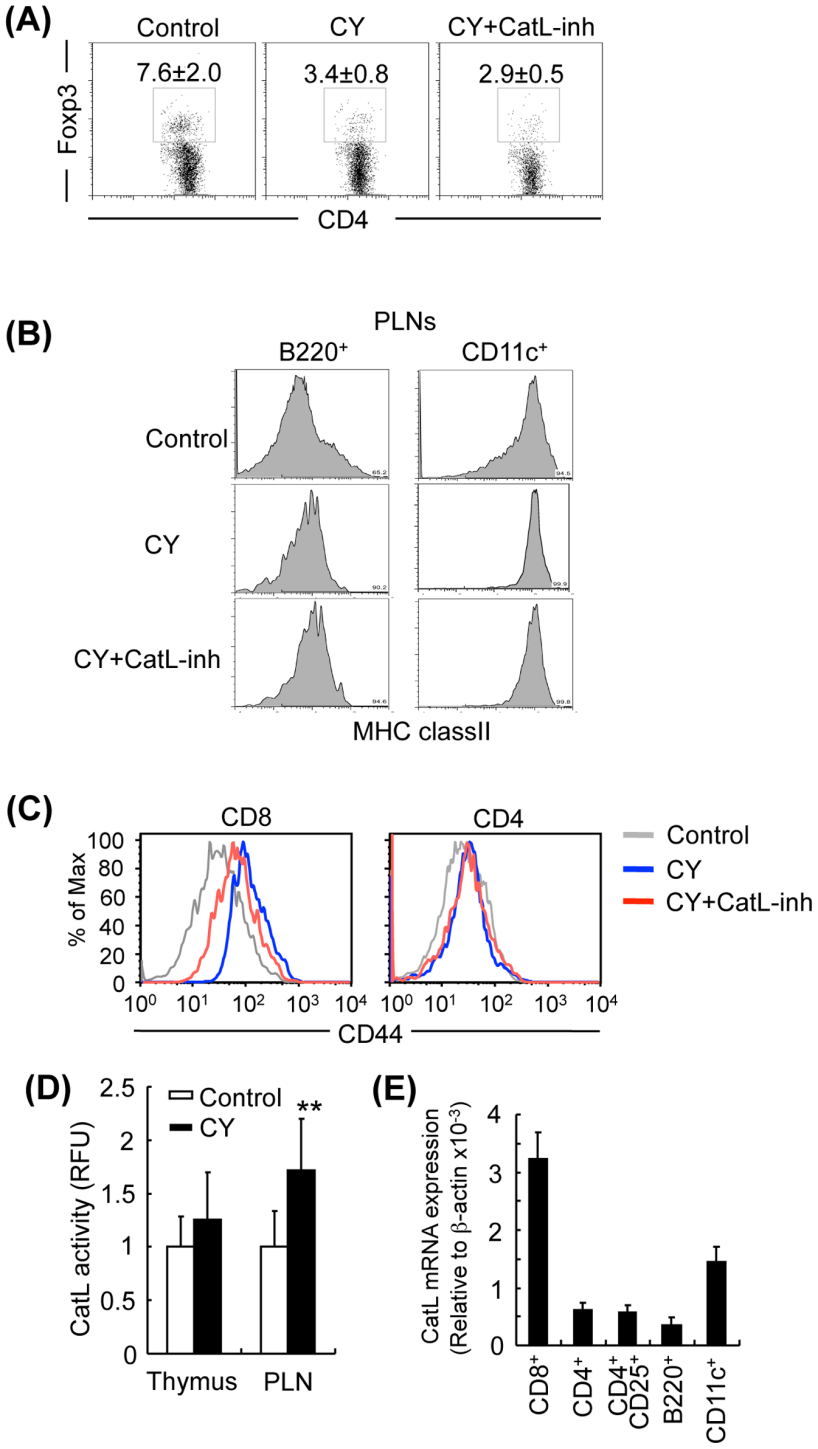
The Effects of CatL-inh Administration on Immune Cells in CY-induced T1D. (A) Flow cytometric analysis was performed to detect intracellular Foxp3 in CD4^+^ T cells of pancreatic lymph nodes (PLNs) from control mice, CY-treated mice and CY+CatL-inh-treated mice. (B, C) CD44 expressions on CD4^+^ and CD8^+^ T cells in PLNs from control, CY-treated, and CY+CatL-inh-treated mice were analyzed by flow cytometry. Results are representative of 3 to 5 mice in each group. (D) Enzymatic activities of cathepsin L in thymus and PLNs from control and CY-treated NOD mice. (E) The mRNA expressions of cathepsin L were detected by real-time PCR using purified CD8^+^, CD4^+^, CD4^+^CD25^+^, B220^+^ and CD11c^+^ cells of PLNs from NOD mice. Data are shown as means ± s.d. of 3 to 4 mice in each group.

It was reported that an increased proportion of Treg cells in the periphery of cahtepsin L-deficient NOD mice was observed compared with that of control NOD mice [10]. When Foxp3^+^CD4^+^ Treg cells of pancreatic lymph nodes (PLN) in CY-treated and control NOD mice were analyzed, the number of Treg cells of PLN from CY-treated NOD mice was significantly reduced compared with control NOD mice as previously reported [13]. On the other hand, there was no increase in the number of Treg cells observed by the administration of CatL-inh (Figure 2A). In addition, to evaluate the effect of *in vivo* treatment with CatL-inh in CY-treated NOD mice on APCs such as B cells and dendritic cells (DCs), the expressions of MHC class II on the cells were detected by flow cytometric analysis. The MHC class II expression on PLN B cells from CY-treated NOD mice was increased compared with that from control NOD mice (Figure 2B). The enhanced level of the expression from CY-treated NOD mice was not changed by the administration of CatL-inh (Figure 2B). Also, there was no difference in the expression level of MHC class II on DCs between CY-treated and CY+CatL-inh-treated NOD mice (Figure 2B). Although cathepsin L is well known to be one of the key proteases in the antigen processing of APCs of the thymus [10], it is still unclear whether cathepsin L plays any role in the peripheral APCs such as B cells and DCs of lymph nodes. To further investigate whether cathepsin L inhibition influences the antigen processing and presentation, an *in vitro* experiment using ovalbumin (OVA)-specific T cell receptor (TCR)-transgenic mice (OT-II) was conducted. In brief, purified CD4^+^ T cells from the spleen of OT-II mice were labeled with carboxyfluorescein diacetate succinimidyl ester (CFSE), and co-cultured with T cell-depleted spleen cells as APCs from B6 mice in the presence of OVA protein with or without CatL-inh or CatS-inh for three days. A small inhibitory effect of CatL-inh (0.05, 0.5 µM) on OT-II T cell response to OVA was observed although inhibitory effect of CatL-inh at high concentration (5 µM) on the T cell response was found (Figure S1). In contrast, CatS-inh more effectively inhibited the proliferative response compared with the effect of CatL-inh, indicating that cathepsin S, rather than cathepsin L, plays a crucial role in antigen processing and presentation of APCs. The *in vivo* experiment using the CatL-inh suggested that there may be no direct effect of cathepsin L on the APCs during the pathogenesis of T1D in NOD mice. Moreover, to examine the effect of CatL-inh administration on T cell activation, CD44 expression, an activation marker, was analyzed using PLN T cells. The enhanced activation of CD8^+^, not CD4^+^ T cells in CY-treated NOD mice was suppressed by the CatL-inh administration (Figure 2C). Furthermore, as shown in Figure 2D, the enzymatic activities of cathepsin L of PLN cells from CY-treated NOD mice was significantly higher than that from control NOD mice. Additionally, we investigated endogenous cathepsin L mRNA levels among various immune cell populations of PLN from NOD mice. The highest expression of cathepsin L mRNA was observed in CD8^+^ T cells (Figure 2E). On the other hand, as shown in Figure 2E and Figure S1, the expression of cathepsin L mRNA of PLN CD8^+^ T cells from NOD mice was significantly higher than that from normal age-matched C57BL/6J mice (B6: 1.28±0.13, NOD: 3.2±0.46×10^−3^ to β-actin mRNA expression, *p*<0.05). By contrast, the expression levels of CD4^+^, CD25^+^CD4^+^, B220^+^ B, and CD11c^+^ cells from NOD mice were similar to the levels of B6 mice (Figure S2). Among the immune cells of B6 mice, the expression levels of CD8^+^ T cells and DCs were considerably increased compared with those of CD4^+^, CD25^+^CD4^+^ and B220^+^ cells (Figure S2). We suggest that this change in the CD8^+^ T cells may account for the protection of diabetes observed in CatL-inh-treated NOD mice.

### Cathepsin L in CD8 T Cells of CY-treated NOD Mice

**Figure 3:**
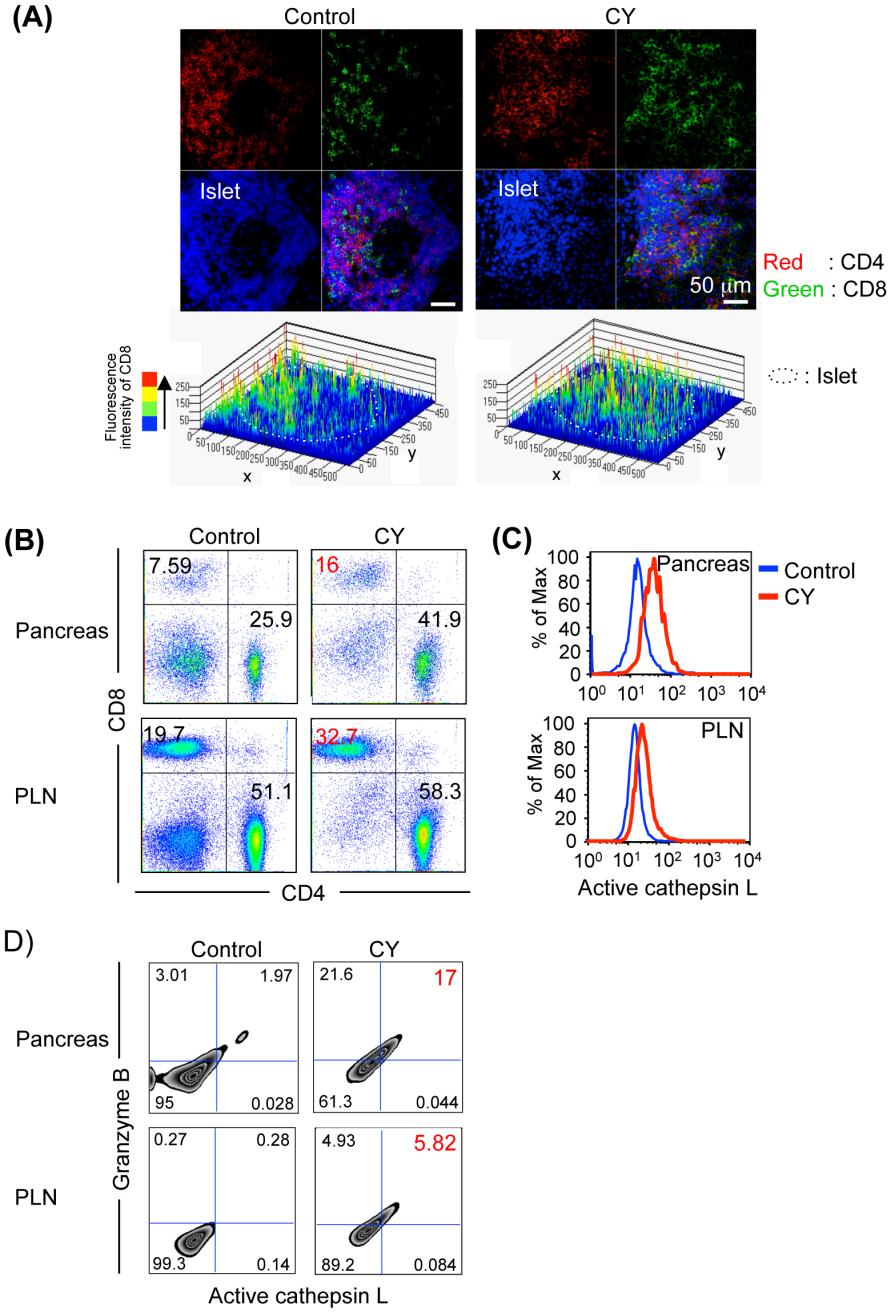
Enzymatic Activity of Cathepsin L of CD8^+^ T cells in T1D. (A) CD4 (red) and CD8 (green) T cells were shown by confocal microscopic analysis. Nuclei were stained with DAPI. Plots depict 2.5 D graphical reconstructions of the green intensity profile of the imaged areas, illustrating individual intensities per pixel utilizing the rainbow scale. (B) CD4^+^ and CD8^+^ T cells on lymphocytes in pancreas tissues and PLNs from control and CY-treated mice were analyzed at one week after the second CY injection by flow cytometry. (C) Enzymatic activities of cathepsin L (Magic Red™) of CD8^+^ T cells were detected by flow cytometric analysis using pancreas tissues and PLNs from control and CY-treated mice. (D) Association of intracellular granzyme B expression with enzymatic activity of cathepsin L in CD8^+^ T cells was evaluated by flow cytometric analysis using pancreas tissues and PLNs from control and CY-treated mice. Results are representative of 3 to 4 mice in each group.

Next, the role of cathepsin L of CD8^+^ T cells in the pancreas tissues from CY-treated NOD mice was analyzed. Immunofluorescence staining showed an increased infiltration of CD8^+^ T cells in the islets from CY-treated NOD mice compared to that from control NOD mice (Figure 3A, right panel). In control NOD mice in which peri-insulitis was observed, the majority of infiltrating lymphocytes was CD4^+^ T cells, plus a small number of CD8^+^ T cells (Figure 3A, left panel). Additionally, flow cytometric analysis indicated that there was a significantly increased number of CD8^+^ T cells as well as CD4^+^ T cells in the pancreas tissues and PLNs from CY-treated NOD mice compared with control NOD mice (Figure 3B). Multiple lines of study have demonstrated the importance of CD8^+^ T cells in the pathogenesis of T1D in NOD mice and T1D patients [16]–[18]. To detect the enzymatic activity of cathepsin L of the infiltrating CD8^+^ T cells in the pancreas tissues, flow cytometric analysis using the fluorogenic substrates (Magic Red™) of cathepsin L was performed. A significantly enhanced cathepsin L activity of pancreatic CD8^+^ T cells from CY-treated NOD mice was observed compared with that from control NOD mice (Figure 3C). In PLNs, a slight increased activity of cathepsin L in CD8^+^ T cells from CY-treated NOD mice was detected (Figure 3C). Moreover, to understand the relationship between cytotoxic activity and enzymatic activity of cathepsin L in CD8^+^ T cells, both granzyme B expression and cathepsin L enzymatic activity were analyzed by flow cytometry. By CY-treatment, the cell numbers of granzyme B^+^ CatL^+^ CD8^+^ T cells in both pancreas and PLNs were significantly increased compared with those from control NOD mice (Figure 3D). These results indicate that cathepsin L might control the cytotoxic activity of CD8^+^ T cells against the islets of pancreas in autoimmune diabetes of NOD mice.

### Effective Inhibition of CatL-inh on CTL Activity of CD8 T Cells

**Figure 4:**
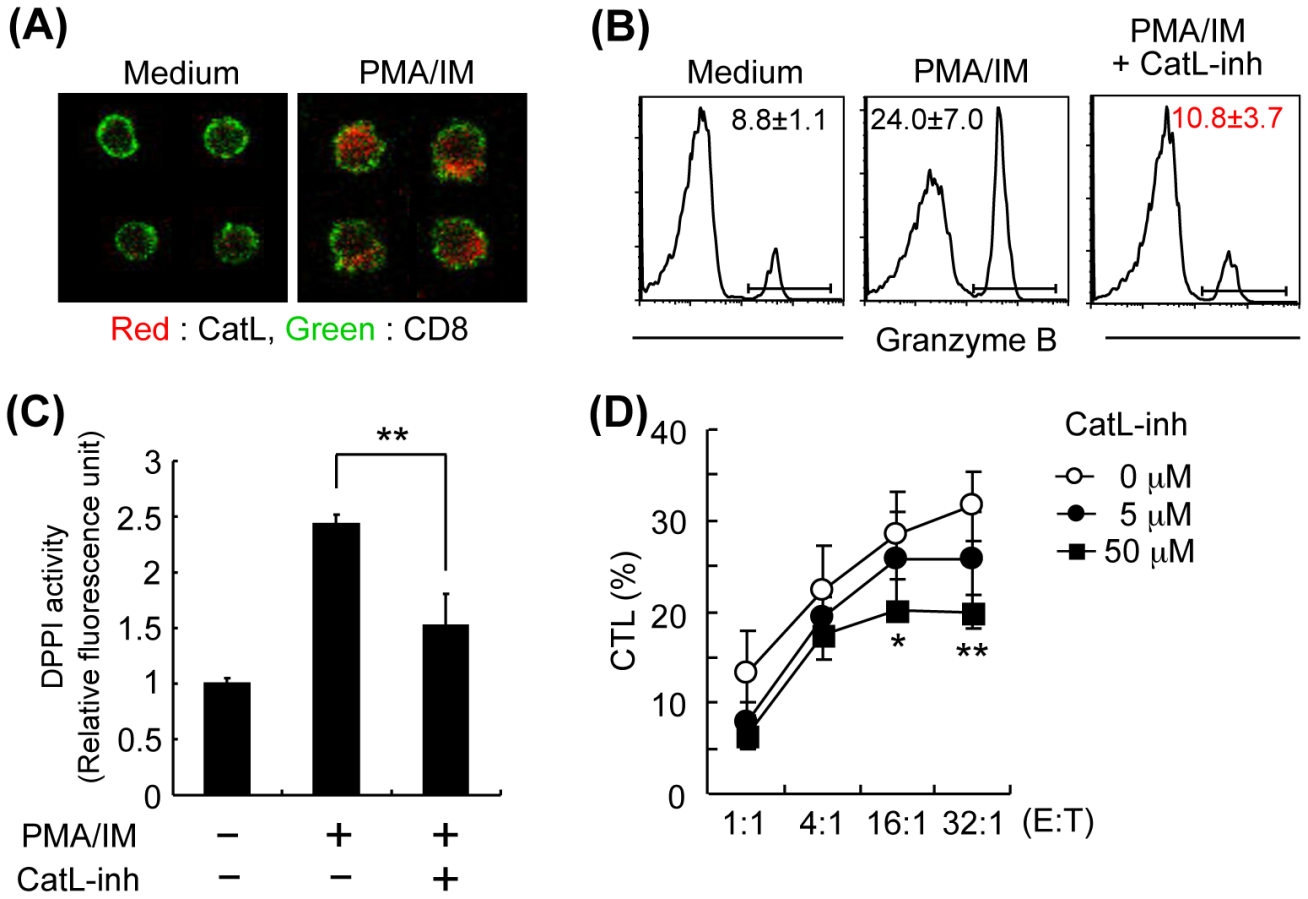
Effect of CatL-inh on Cytotoxic CD8^+^ T Cells. (A) Enzymatic activity of cathepsin L of activated CD8^+^ T cells from NOD mice was detected by confocal microscopic analysis. Purified CD8^+^ T cells were stimulated for 24 hours with PMA and ionomycin and then stained with Magic Red™ (red) and FITC-conjugated anti-CD8 mAb (green). Original magnification, 630×. (B) Granzyme B expressions of CD8^+^ T cells stimulated with PMA and ionomycin for 24 hours in the presence of CatL-inh were detected by flow cytometric analysis. (C) DPPI enzymatic activities of CD8^+^ T cells from NOD mice were measured. PMA/ionomycin-stimulated CD8^+^ T cells were incubated with or without CatL-inh. The activities were shown as relative fluorescence units to the negative control samples. (D) Cytolytic activity of CD8^+^ T cells against enriched islet cells were evaluated by ^51^Cr release assay. PMA/ionomycin-stiumlated CD8^+^ T cells were incubated with ^51^Cr-labeled target cells for 6 hours in the presence of CatL-inh (0, 5, and 50 µM). E:T, effector/target. Data are shown as means ± s.d. of triplicate wells. *, *P*<0.05, and **, *P*<0.01.

To further investigate the cellular mechanism of CD8^+^ T cell activation through cathepsin L, *in vitro* experiments using purified CD8^+^ T cells from PLNs of NOD mice were performed. No toxic effect of CatL-inh on CD8^+^ T cells in this experiment was observed (Figure S3). When the CD8^+^ T cells were stimulated with phorbol 12-myristate 13-acetate (PMA) and ionomycin (IM), the increased level of enzymatic activity of cathepsin L was confirmed by confocal analysis using Magic Red™ (Figure 4A). Increased granzyme B expression of CD8^+^ T cells was observed with the addition of PMA/IM. Then the enhanced expression level was decreased by the addition of CatL-inh (Figure 4B). It has been reported that dipeptidyl peptidase I (DPPI) is a granule protease that plays a requisite role in processing the proenzyme form of the cytotoxic T-lymphocyte (CTL) granule serine proteases (granzymes) [19]–[22]. In addition to its role in the lysosomal protein degradation, DPPI functions as a key enzyme in the activation of granule serine peptidases in CTL [23]. Since it has been demonstrated that cathepsin L could be an important activator of DPPI from its precursor form *in vitro* [24], the enzymatic activity of CD8^+^ T cells from NOD mice was examined. DPPI enzymatic activity of CD8^+^ T cells was elevated by stimulation of PMA/IM, and the addition of CatL-inh reduced the increased DPPI activity (Figure 4C). Finally, it was examined whether cytotoxicity of the activated CD8^+^ T cells against the enriched islet cells is suppressed by CatL-inh. The islet cells from pancreas of NOD mice were isolated as described in Methods. Over 70% of the enriched islet cells produced insulin (Figure S4A). To evaluate the toxic effect of CatL-inh on the islet cells as target cells, the isolated islet cells were incubated with CatL-inh for 24 hours. No change of apoptotic or necrotic cells showing annexin-V and PI was observed by this incubation (Figure S4B). CTL activity of CD8^+^ T cells against the target cells from pancreas of NOD mice was significantly suppressed by CatL-inh (Figure 4D). Theses results indicated that a simple mechanism of inhibiting the activities of DPPI by CatL-inh could provide a powerful way to regulate the ability of immune effector CD8^+^ T cells to kill their targets.

### Effective Therapy of CY-induced T1D by Small Interference (si) RNA Targeting Cathepsin L Gene

**Figure 5:**
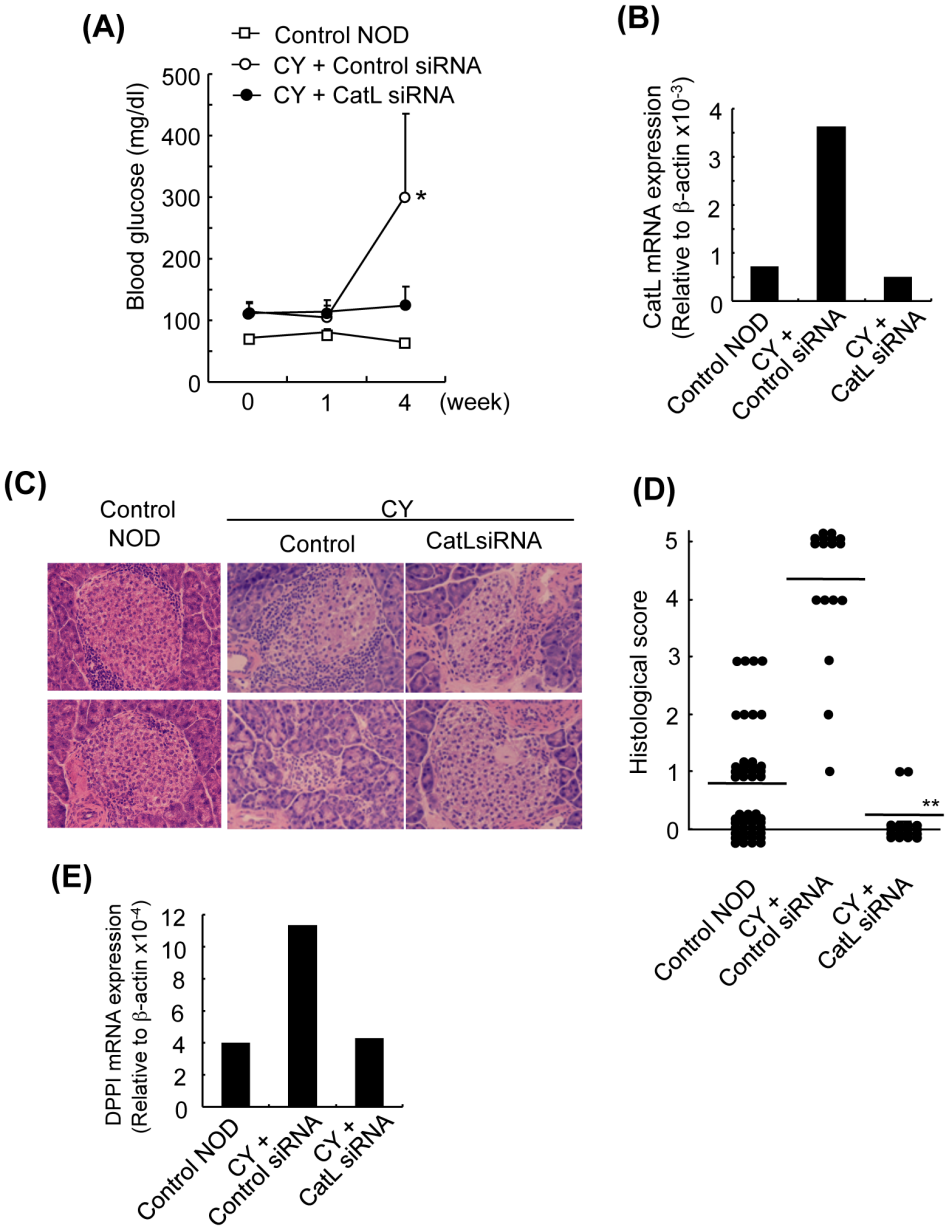
*In Vivo* Therapeutic Effects of Cathepsin L Gene Targeting on T1D. (A) CY-treated NOD mice were administered with control siRNA or CatL siRNA. The blood glucose level was measured weekly. (B) Cathepsin L mRNA expressions of PLN CD8^+^ T cells were detected by real-time PCR. (C) Histopathological analysis of pancreas tissues was shown. Results are representative of 4 or 5 mice in each group. (D) Histological score of islets from control (n = 4), control siRNA (n = 5) or CatL siRNA-treated (n = 5) NOD mice. Data are shown as the histological score of individual islets. (E) Expression of CD44 on CD8^+^ T cells was analyzed by flow cytometry. The results are representative of three mice in each group. (E) DPPI mRNA expressions of CD8^+^ T cells in PLNs were analyzed by real-time PCR. Results are representative of three independent experiments.

To further confirm the effects of cathepsin L-inhibition, it was examined whether injection of athelocollagen-mediated small interference (si) RNA for cathepsin L gene silencing provides long-term suppression of diabetes development in CY-treated NOD mice. Evidence was obtained demonstrating that application of siRNA targeting cathepsin L (CatL siRNA) intraperitoneally (i.p.) caused a marked decrease in the development of CY-induced diabetes in NOD mice showing a high level of blood glucose (Figure 5A). To confirm the effect of the Cat L-siRNA on the expression of the target gene, mRNA expression of cathepsin L of PLN CD8^+^ T cells was quantified by real-time PCR. A reduced expression of cathepsin L mRNA from CatL siRNA-treated mice was observed compared with that from control siRNA-treated mice (Figure 5B). Histological analysis showed that inflammatory infiltration of lymphocytes and destructive changes in islets of the pancreas tissues from CatL siRNA-treated mice were clearly prevented in contrast to the severe inflammatory lesions of control siRNA-treated mice (Figure 5C). Furthermore, the histological score of infiltrates in islets from CatL siRNA-treated mice was significantly reduced compared with that from control siRNA-treated mice (Figure 5D). There were no differences in the cell numbers of T cell populations of PLNs between CatL siRNA- and control siRNA-treated mice (Figure S5). In addition, the expression level of DPPI mRNA of PLN CD8^+^T cells from CatL siRNA-treated mice was lower than that from control siRNA-treated mice (Figure 5E). These results demonstrated that the gene targeting of cathepsin L by *in vivo* administration of siRNA may be one therapeutic strategy for autoimmune diabetes.

### Cathepsin L mRNA Expression in Spontaneous T1D of NOD Mice

**Figure 6:**
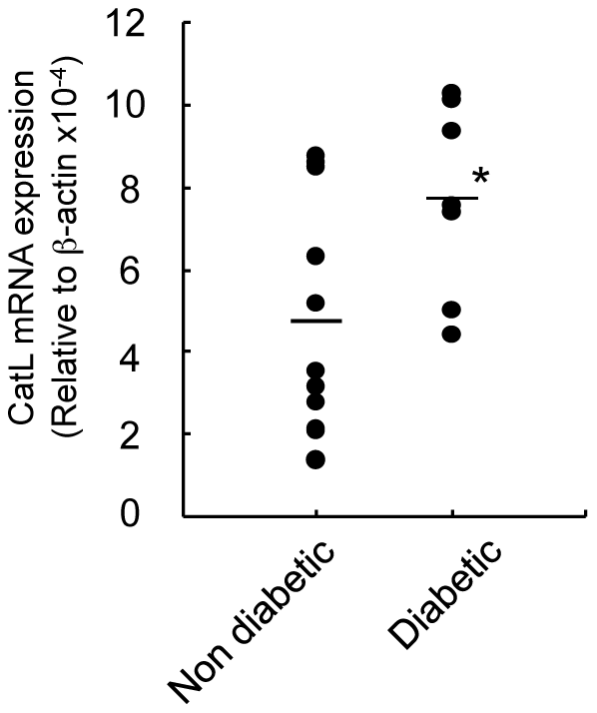
Cathepsin L Expression of CD8^+^ T Cells of NOD Mice. The mRNA expressions of cathepsin L of purified CD8^+^ from PBLs from non-diabetic and diabetic NOD mice were analyzed by real-time PCR. Mice exceeding 250 mg/dl blood glucose were considered diabetic. Data are shown as means ± s.d. of 5 to 10 mice in each group *, *P*<0.05.

However, it is still unclear whether cathepsin L has an effect on the onset of autoimmne diabetes in NOD mice. It is well known that a considerable proportion of non-diabetic mice are included among NOD littermates [25], [26]. Thus, we analyzed the cathepsin L mRNA levels of purified CD8^+^ T cells from peripheral blood lymphocytes (PBLs) of non-diabetic and diabetic NOD mice from three to nine months of age. As shown in Figure 6, the expression of cathepsin L mRNA of CD8^+^ T cells from diabetic NOD mice was significantly higher than that from non-diabetic NOD mice. However, there was a range of cathepsin L expressions even in non-diabetic mice. Some differences in the disease development or onset in NOD littermates may be associated with expression levels of cathepsin L. By contrast, enhanced expression of granzyme B in diabetic NOD mice compared with that in non-diabetic NOD mice could not be found (data not shown). On the other hand, when we compared the granzyme B expressions of infiltrating CD8^+^ T cells in the pancreas tissues between non-diabetic and diabetic NOD mice by flow cytometry, significantly higher granzyme B expression of diabetic pancreatic CD8^+^ T cells was observed in diabetic NOD mice (Figure S6). These findings suggest that cathepsin L expression in CD8^+^ T cells of peripheral blood dose not strictly reflect the pathogenesis of autoimmune diabetes in NOD mice, but it may be one of helpful tools for diagnosis of T1D as well as the effective strategy by targeting cathepsin L gene.

## Discussion

Although type-1 diabetes can be controlled by insulin injections, individuals who develop the disease may suffer long-term complications that include blindness, kidney failure and premature vascular disease leading to early death [27], [28]. The disease is increasing in incidence worldwide. Thus there is a pressing need to understand pathogenic mechanisms to design effective immunotherapeutic preventions. These results demonstrate that cathepsin L, which has been thought to be an antigen-processing enzyme, plays a critical role in the peripheral immune system.

Cathepsin L functions under the acidified conditions in the endosome, and plays an important role in the DPPI signaling events in CD8^+^ T cells. It is well known that cathepsin L is involved in the proteolysis of MHC class II-associated invaliant chain (Ii) and the antigen presentation in cortical thymic epithelial cells [29]–[31]. In this study, it was demonstrated that cathepsin L has a pivotal effect at the upstream of granzyme B on cytotoxicity of CD8^+^ T cells in the development of T1D other than the maintenance of Treg cells in the periphery and the thymic selection. Two reports using cathepsin L-deficient mice focused on Treg cells which control autoimmune diabetes in NOD mice [10], [32]. Both papers showed that the expression of cathepsin L was not required for the disease induction. However, there was a big difference in the incidences of cumulative diabetes between CD25-depleted control and CD25-depleted *Cat-L* ^−/−^ splenocytes-transferred recipients [10], [32]. We speculate that Treg cells play an important role in the disease induction or onset of autoimmune diabetes in NOD mice, and that the development or severity of the disease may be attributed to other immune cells including CD8^+^ T cells in addition to the Treg cells. Moreover, although significantly increased Treg cells in cathepsin L-deficient NOD mice were detected, there was no difference in the Treg cell number of PLNs between CY and CY+CatL-inh-treated NOD mice in our experiment. As for the discrepancy, the cathepsin L inhibitor might be effective for peripheral T cells, but not T cell differentiation including Treg cells in the thymus. The effectiveness may depend on the dose, time span, or timing of CatL-inh administration, while the weakness of the treatment with specific CatL-inh is that the metabolic time is considerably short *in vivo*. Within approximately 24 hours, the effectiveness had disappeared in the animal's bodies. In addition, it was reported that there was an effect of cathepsin L inhibition using CatL-inh on antigen processing in the peripheral lymphoid tissues [33]. In our study using CatL-inh and siRNA of cathepsin L, although we focused on CD8^+^ T cells, possible roles in other immune cells besides CD8^+^ T cells should be researched.

As for the adverse effects of injection of cahtepsin L inhibitor, when body weight, health condition, and a histopahtological examination of all organs from CatL-inh-administerred mice was checked, there was no significant change. This suggests that the CatL-inh has no toxicity or side effects, and the application may be clinically useful. Also the administration of siRNA targeting cathepsin L genes using athelocollagen may have a temporary effect on the peripheral T cells. It is possible that the siRNA might not have any effect on the gene-knockdown in the thymus. When atelocollagen, which is positively charged, interacts with the negatively charged siRNA duplex, a siRNA/atelocollagen complex is formed. This is a nanosize particle with a diameter of 100–300 nm. The siRNA/atelocollagen complex was highly stable against nucleases and a prolonged release of genes and oligonucleotides without the digestion of RNase [34]. Additionally, it was confirmed that regarding to specificity for cathepsin L siRNA, there was a reduction of cathepsin L mRNA of CD8^+^ T cells in PLN from cathepsin L siRNA-treated NOD compared with that from control NOD mice (relative expression to β-actin mRNA; control siRNA: 3.62×10^−3^, cathepsin L siRNA: 0.48×10^−3^).

A previous report showed that a reduced number of CD4^+^ T cells in the thymus and periphery of cathepsin L deficient mice was observed, and that CD8^+^ T cells in the deficient mice were relatively increased [29]. Cathepsin L was found to be necessary for invariant chain (Ii) degradation in cortical thymic epithelial cells [29]. The degradation of Ii is known to be a critical step in MHC class II-restricted antigen presentation that is closely related with CD4^+^ T cell differentiation [35], [36]. In addition to the function of cathepsin L in central tolerance of thymus, we propose that a significantly upregulated cathepsin L activity in cytotoxic CD8^+^ T cells from NOD mice might play a critical role in the pathogenesis of T1D. It is known that CD8^+^ T cells as well as CD4^+^ T cells are pivotal effector cells for rapid destruction of pancreatic b-cells in CY-induced T1D [37]. In our experiment, the cytotoxic activity of NOD CD8^+^ cells from spleen against enriched islet cells from NOD pancreas was significantly reduced by the addition of CatL-inh. Splenic CD8^+^ T cells including a very low number of b cell antigen-specific CD8^+^ T cells in NOD mice were stimulated with PMA and ionomycin, and used for *in vitro* CTL assay. The antigen-specificic CTL activity of CD8^+^ T cells in development of autoimmune diabetes of NOD mice remains unsolved.

On the other hand, it would be helpful for diagnosis of spontaneous diabetes in NOD mice to measure cathepsin L expression in the peripheral T cells. Under three months of age, there was no change in the cathepsin L expression. In addition, we have confirmed that the expression of cathepsin L mRNA of CD8^+^ T cells in peri-pancreatic LN was significantly enhanced compared with that of the other immune cells including CD4^+^T cells, Treg cell, B cells, and dendritic cells. Those findings suggested that cathepsin L in CD8^+^ T cells might be activated at an advanced stage of the disease in NOD mice. However, it is still unclear why cytotoxic activity of CD8^+^ from NOD mice could be upregulated via the axis of cathepsin L, DPPI, and granzyme B. In addition, we should pay attention for using the mRNA level of cathepsin L in the peripheral CD8^+^ T cells as the level dose not strictly reflect the onset or development of autoimmune diabetes in NOD mice. Further investigation would be required for the clinical application in human patients.

Foxp3-expressing CD4^+^ thymus-derived naturally occurring Treg cells play an indispensable role in the maintenance of self-tolerance and immune homeostasis [38], [39]. They are potent suppressors of organ-specific autoimmunity such as T1D, inflammatory bowel disease, and autoimmune gastritis [40]. Multiple reports have implicated Treg cells in the prevention of T1D [41], [42]. In aged NOD mice, T1D resistance has been correlated with the expansion of Treg cells with regulatory activity within inflamed pancreatic lymph nodes [43]. Furthermore, it was demonstrated that Foxp3-deficient NOD mice, which are deficient in Treg cells, display an increased incidence and earlier onset of T1D compared with control NOD mice [44]. Administration of high-dose CY to prediabetic NOD mice leads to rapid synchronous onset of T1D (11), and transfer of lymphocytes from syngenic non-diabetic NOD mice after CY treatment protects from T1D onset [12]. As for a cellular mechanism for CY-induced T1D in NOD mice, it was reported that the onset of CY-induced T1D is associated with a reduction of Treg cells [13]. It is possible that a reduction of Treg cells in NOD mice by CY injection might trigger up-regulated cathepsin L activity following cytotcoxicity of CD8^+^ T cells against pancreatic b-cells through unknown molecular mechanisms.

Our previous report indicated that cathepsin S inhibitor can prevent Sjogren' syndrome which is one of the organ-specific autoimmune diseases found in salivary and lacrimal glands [8]. Moreover, it was demonstrated that impairment of Ii degradation and diminished collagen-induced arthritis were observed in cathepsin S-deficient mice [45]. In the case of a cathepsin S inhibition or deficiency, the protection of autoimmune response seems to be due to modulation of antigen presentation. In the present study, there was no observation of therapeutic effect of cathepsin S inhibitor on CY-induced T1D in NOD mice. This implies that lysosomal proteases such as cathepsin L and S play a critical role in each step of pathogenesis for autoimmune diseases via complex molecular mechanisms. Careful attention should be paid to the therapeutic effects of cathepsin L inhibition on the immune system in the treatment of autoimmune diabetes, the pathogenesis of which is dependent on CD8^+^ T cells. Targeting these CD8^+^ T cells using cathepsin L inhibitor could be a strategy for the prevention and cure of diabetes in the near future.

Taken together, the results of the experiments presented in this report show that treatment with cathepsin L inhibition prevents the cytotoxic CD8^+^ T cell response in autoimmune diabetes NOD mice via inhibiting granzyme B activation. These data point to cathepsin L as a novel regulatory mechanism of cytotoxicity, suggesting a potential use of cathepsin L inhibition including siRNAs delivery as a new therapeutic target in autoimmune diabetes.

## Materials and Methods

### Ethics

This study was conducted according to the principles expressed in the Declaration of Helsinki. The study was approved by the Institutional Review Board of the University of Tokushima (toku09021).

### Mice and Treatment

NOD mice were reared in our specific pathogen-free mouse colony and given food and water ad libitum. Experiments were humanely conducted under the regulation and permission of the Animal Care and Use Committee of the University of Tokushima, Tokushima, Japan. Prediabetic (7 to 8-weeks-old) female NOD mice were intraperitoneally injected twice with 4 mg cyclophosphamide (CY).

### Cathepsin Inhibitors

Specific inhibitors for cathepsin B (CA074), cathepsin L (Clik148 and Clik195), and cathepsin S (Clik60) were developed with the help of computer-graphic modeling based on the stereo-structure [46]. We administered the cathepsin inhibitors after the second injection of CY. In detail, 7 to 8-weeks-old prediabetic NOD mice were intraperitoneally injected with CY (4 mg per mouse) once per week for two weeks. At 9 to 10-weeks-old, cathepsin inhibitors were intraperitoneally administered (0.1 mg per mouse) every two days for four weeks. We sacrificed the mice for analysis at 13 to 14-weeks-old.

### Assessment of Diabetes

The blood glucose levels and the urine glucose were monitored weekly with a Glucometer (Kodama, Tokyo, Japan) using 50 µl blood from the tail vein, and with Keto-Diastix (Bayer-Sankyo Co., Ltd., Tokyo, Japan), respectively.

### Histology

All organs were removed from the mice, fixed with 4% phosphate-buffered formaldehyde (pH 7.2) and prepared for histological examination. The sections were stained with hematoxylin and eosin (H&E). For semiquantitative evaluation of infiltration, histological analysis using sections containing five or more islets was performed as previously described [47]. In brief, the degree of cellular infiltration was scored from 0 to 5 as follows: 0 = no inflammation; 1 = infiltrates in small foci at the islet periphery; 2 = infiltrates surrounding the islets (peri-insulitis); 3 = intraislet infiltration <50% of the islet, without islet derangement; 4 = extensive infiltration,  = 50% of the islet, cell destruction and prominent cytoarchitectural derangement; 5 = islet atrophy because of β cell loss. The evaluation was carried out by three pathologists in a blinded manner.

### Flow Cytometric Analysis

Surface markers on lymphocytes in pancreatic lymph nodes (PLNs) and pancreas were identified by mAbs with BD FACSCant flow cytometer (Beckman Coulter, Inc., Miami, FL). Rat mAbs to FITC-, PE-conjugated anti-CD8 and CD4 mAbs (eBioscience, San Diego, CA) were used. For detection of T cell activation markers, PE-Cy5-conjugated anti-CD44 mAbs (eBioscience) were used. Intracellular Foxp3 and granzyme B (eBioscience) expressions with an intracellular Foxp3 detection kit (eBioscience) were analyzed according to the manufacturer's instructions. Enzymatic activities of cathepsin L were assessed using Magic Red™ cathepsin detection kit for cathepsin L (Immunochemistry Technologies, LLC, Bloomington, MN). Briefly, lymphocytes were incubated with Magic Red™ fluorogenic substrates of cathepsin L according to the manufacturer's instructions. The red fluorescence was detectable as a result of intracellular enzymatic cleavage. The data were analyzed with FlowJo FACS analysis software (Tree Star).

### Cathepsin L Activity Detection Assay

Cathepsin L activity was assayed in the thymus and PLNs from CY-treated and control NOD mice using a cathepsin L activity assay kit (BioVision, Mountain View, CA). Briefly, 10 µg cytoplasmic lysates of thymus and PLNs were incubated with cathepsin L substrates labeled with amino-4-trifluoromethyl coumarin (AFC) at 37°C for an hour. The AFC cleaved by cathepsin L was read with a fluorometric microplate reader (Tecan, Crailsheim, Germany) at excitation and emission wavelengths of 400 nm and 505 nm.

### Real-time Quantitative Reverse-transcription-polymerase Chain Reaction (Real-time PCR)

CD8^+^ cells, CD4^+^ cells, CD4^+^CD25^+^ cells, B220^+^ cells and CD11c^+^ cells were prepared from PLNs by positive selection using anti-CD8, CD4, CD25, B220 (eBioscience) and CD11c (MBL International, Woburn, MA) antibodies, and magnetic beads (Dynal Biotech, Oslo, Norway). Total RNA was extracted using TRIzol reagent (Invitrogen, Carlsbad, CA) and reverse transcribed. Transcript levels of cathepsin L, DPP1 and β-actin were detected using PTC-200 DNA Engine Cycler (BioRad) with SYBR Premix Ex Taq (Takara, Kyoto, Japan). Primer sequences were as follows: cathepsin L: forward, 5′-GTGGACTGTTCTCACGCTCA-3′ and reverse, 5′-TATCCACGAACCCTGTGTCA-3′, DPPI: forward, 5′-TCTGTCAATGAGTGAGCTGTGTCAA-3′ and reverse, 5′-TGCGCTCATGTGTGTATGGAAG-3′ and b-actin: forward, 5′- AAATCTGGCACCACACCTTC -3′ and reverse, 5′- AGAGGCGTACAGGGATAGCA -3′.

### Confocal Microscopic Analysis

The frozen sections of pancreas tissues from control and CY-treated NOD mice were fixed with cold acetone, blocked with M.O.M.™ blocking reagent (Vector Laboratories, Inc., Burlingame, CA), and then stained with the antibodies FITC-conjugated antibody to CD8 (eBioscience) and biotinylated antibody to CD4 (BioLegend, San Diego, CA) for over night. After washes in PBS, the sections were stained with Alexa Fluor 488 goat anti-FITC IgG and Alexa Fluor 568-conjugated streptavidin (Invitrogen, Carlsbad, CA) for 40 minutes. The nuclear DNA was stained with 4′,6-diamino-2-phenylindole dihydrochloride (DAPI) (Invitrogen). The sections were visualized with a laser scanning confocal microscope (Carl Zeiss) at a magnification of 400× or 630×. Quick Operation Version 3.2 (Carl Zweiss) for imaging acquisition and Adobe Photoshop CS2 (Adobe System) for image processing was used.

### Cell Purification

For purification of CD8^+^ subset, lymphocytes in pancreas and PLNs were treated for 30 min at 4°C with mAbs specific for CD4, CD25, CD11c, NK1.1, B220, and MHC class II (eBioscience), and then CD8^+^ T cells were isolated by negative selection with magnetic beads (Dynal).

### Culture Conditions

Cells were cultured in RPMI 1640 containing 10% FCS, L-glutamine, penicillin/streptomycin and 2-mercaptoethanol in 96-well round-bottom microtiter plates stimulated with (0.5∼5 ng/ml) PMA and 500 ng/ml ionomycin (SIGMA, St. Louis, MO).

### *In Vitro* Cathepsin L Activity Detection Assay

Cathepsin L activity of CD8^+^ T cells stimulated with PMA and ionomycin (IM) was assessed by confocal microscopic analysis using a Magic Red™ cathepsin detection kit. In brief, CD8^+^ T cells were stimulated with PMA and IM for 24 hours. Cells were stained with Magic Red™ according to the manufacture's instructions. After staining, cells were deposited onto poly-L-lysine-coated glass slides and were fixed with 0.25 to 1% paraformaldehyde. Subsequently, cells were stained with anti-CD8 mAb and visualized with a laser scanning confocal microscope. A 63×1.4 oil DIC objective lens was used.

### DPPI Activity Assay

DPPI activity was assayed by the liberation of the fluorescent leaving group, 7-amino-4-ethyl coumarin (AMC), and measured as increase in fluorescence using a fluorometer. Briefly, purified CD8^+^ T cells were stimulated with PMA/IM in the presence of cathepsin inhibitor (10^−6^ M) for 40 hours. Cell extracts were added to H-Gly-Arg-AMC in assay buffer (25 mM MES, 50 mM NaCl, 5 mM DTT, pH 6.0) and incubated for 1 hour at 37°C. After incubation, substrate cleavage was measured by fluorophotometer at excitation and emission wavelengths of 380 nm and 460 nm.

### Cytotoxic Assay

For target cells, the enriched islets were obtained from NOD mice at 4-weeks-old as previously described [48], [49]. Purified CD8^+^ T cells as effector cells in spleen from NOD mice were stimulated with PMA/IM in the presence of cathepsin L inhibitor (0, 5, and 50 µM) for 24 hours. Maximum cytotoxicity was induced by 0.1% Triton X. ^51^Cr (Perkin Elmer, Wellesley, MA)-labeled target cells were incubated with the effector cells for 6 hours at the indicated E:T ratios. ^51^Cr release of the supernatants was measured with a g-scintillation counter (Aloka, Tokyo, Japan). Corrected % lysis was calculated as: corrected % lysis = 100× (test ^51^Cr released – spontaneous ^51^Cr released)/(maximum ^51^Cr released - spontaneous ^51^Cr released).

### *In Vivo* Treatment with Cathepsin L siRNA

Small interfering RNA (siRNA) of cathepsin L and negative control (B-bridge International, Inc., Sunnyvale, CA) were used for analysis of *in vivo* therapeutic effects on the T1D in CY-treated NOD mice. Sequences of the oligonucleotide were as follows: cathepsin L: 5′-GAGCGAUAUGGGAGAAGAATT-3′ and negative control: 5′-ATCCGCGCGATAGTACGTA-3′. Briefly, equal volumes of atelocollagen [50] (Koken, Tokyo, Japan) were combined with siRNA solution and mixed by rotating at 4°C for 20 mins. The cathepsin L siRNA/atelocollagen complexes or negative control siRNA/atelocollagen complexes were i.p. administered into CY-treated NOD mice at doses of 5 nmol/mouse/twice a week.

### Statistical Test

The Student' *t* test was used for statistical analysis. Values of *p*<0.05 were considered as significant.

## Supporting Information

Figure S1 (A) Effect of Cat-L and S inhibitors on antigen processing and presentation. Splenic CD4+ T cells from OVA-specific T cell receptor transgenic mice (OT-II) were labeled with carboxyfluorescein diacetate succinimidyl ester (CFSE), and co-cultured with T cell-depleted spleen cells as APCs from B6 mice in the presence of OVA protein with or without CatL-inh or CatS-inh for 3 days. (B) Proliferation to OVA was evaluated by divided cells (%). Data are shown as means ± s.d. of triplicate wells *, P<0.05, **, P<0.01. Results are representative of two independent experiments.
(0.39 MB TIF)

Figure S2 The mRNA expressions of cathepsin L in PLN cells from C57BL/6 mice. The mRNA expressions of cathepsin L in purified CD8+ T cells, CD4+ T cells, CD25+CD4+ T cells, B220+ B cells and CD11c+ dendritic cells of PLNs from C57BL/6 mice were detected by real-time PCR. Data are shown as means ± s.d. of triplicate wells, and representative of three mice.
(0.20 MB TIF)

Figure S3 Evaluation of toxic effect of cathepsin L inhibitor on CD8+ T cells. CD8+ T cells from NOD mice were incubated with CatL-inh (10-5 M) for 24 hours. Apoptotic or necrotic cells were detected with annexin-V and propidium iodide (PI) staining by flow cytometry. Results are representative of three independent experiments.
(0.63 MB TIF)

Figure S4 The toxic effect of CatL-inh on enriched pancreatic islet cells. (A) The islet cells were enriched from the pancreas of NOD mice, and then stained with anti-insulin mAb (HyTest Ltd. Turku, Finland) and Alexa568-conjugated mouse IgG (H+L) as the secondary antibody. The nuclei were stained with DAPI. Insulin-producing cells (red) were detected by confocal microscopic analysis. Results are representative of two independent experiments. (B) The islet cells were incubated with CatL-inh (0, 10-6, and 10-5 M) for 24 hours. Apoptotic or necrotic cells were detected with annexin-V and propidium iodide staining by flow cytometry. Results are representative of three independent experiments.
(0.78 MB TIF)

Figure S5 The population of PLN cells from the mice administered with control siRNA or CatL siRNA. The graph shows the mean frequency of CD8+ T cells, CD4+ T cells, B220+ B cells and CD11c+ dendritic cells. Data are shown as means ± s.d. of 3 mice.
(0.21 MB TIF)

Figure S6 Granzyme B expression of pancreatic CD8+ T cells from non-diabetic and diabetic NOD mice was analyzed by flow cytometric analysis. The result is representative of two independent experiments.
(0.47 MB TIF)
