# Supplementary figures and images for: Cathepsin L Inhibition Prevents Murine Autoimmune Diabetes via Suppression of CD8+ T Cell Activity
Source: PLoS One. 2010 Sep 22;5(9):e12894. doi: 10.1371/journal.pone.0012894 (PMC2943924; doi:10.1371/journal.pone.0012894)

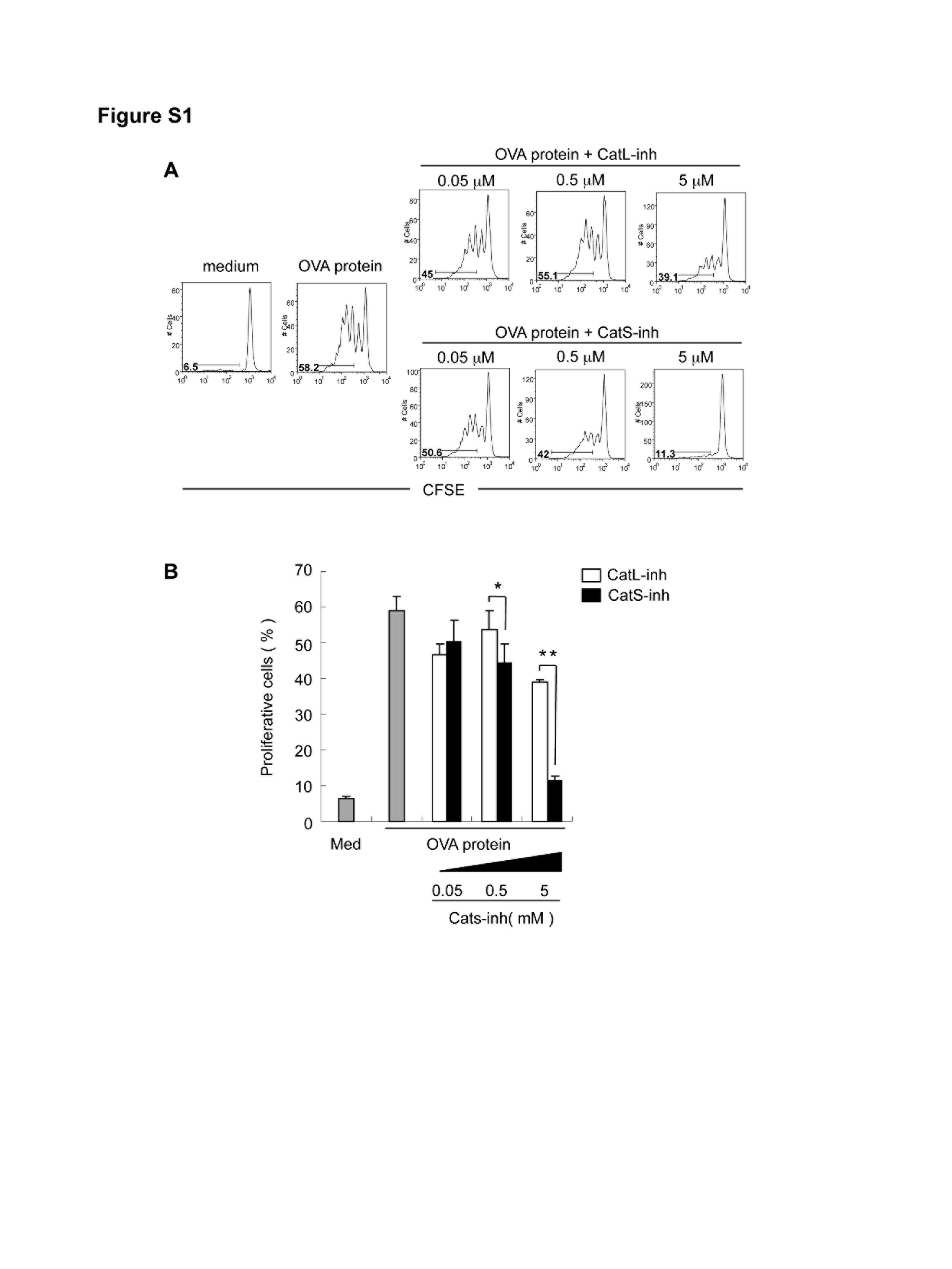

Supplement: Figure S1 — (A) Effect of Cat-L and S inhibitors on antigen processing and presentation. Splenic CD4+ T cells from OVA-specific T cell receptor transgenic mice (OT-II) were labeled with carboxyfluorescein diacetate succinimidyl ester (CFSE), and co-cultured with T cell-depleted spleen cells as APCs from B6 mice in the presence of OVA protein with or without CatL-inh or CatS-inh for 3 days. (B) Proliferation to OVA was evaluated by divided cells (%). Data are shown as means ± s.d. of triplicate wells *, P<0.05, **, P<0.01. Results are representative of two independent experiments. (0.39 MB TIF) [file pone.0012894.s001.tif]

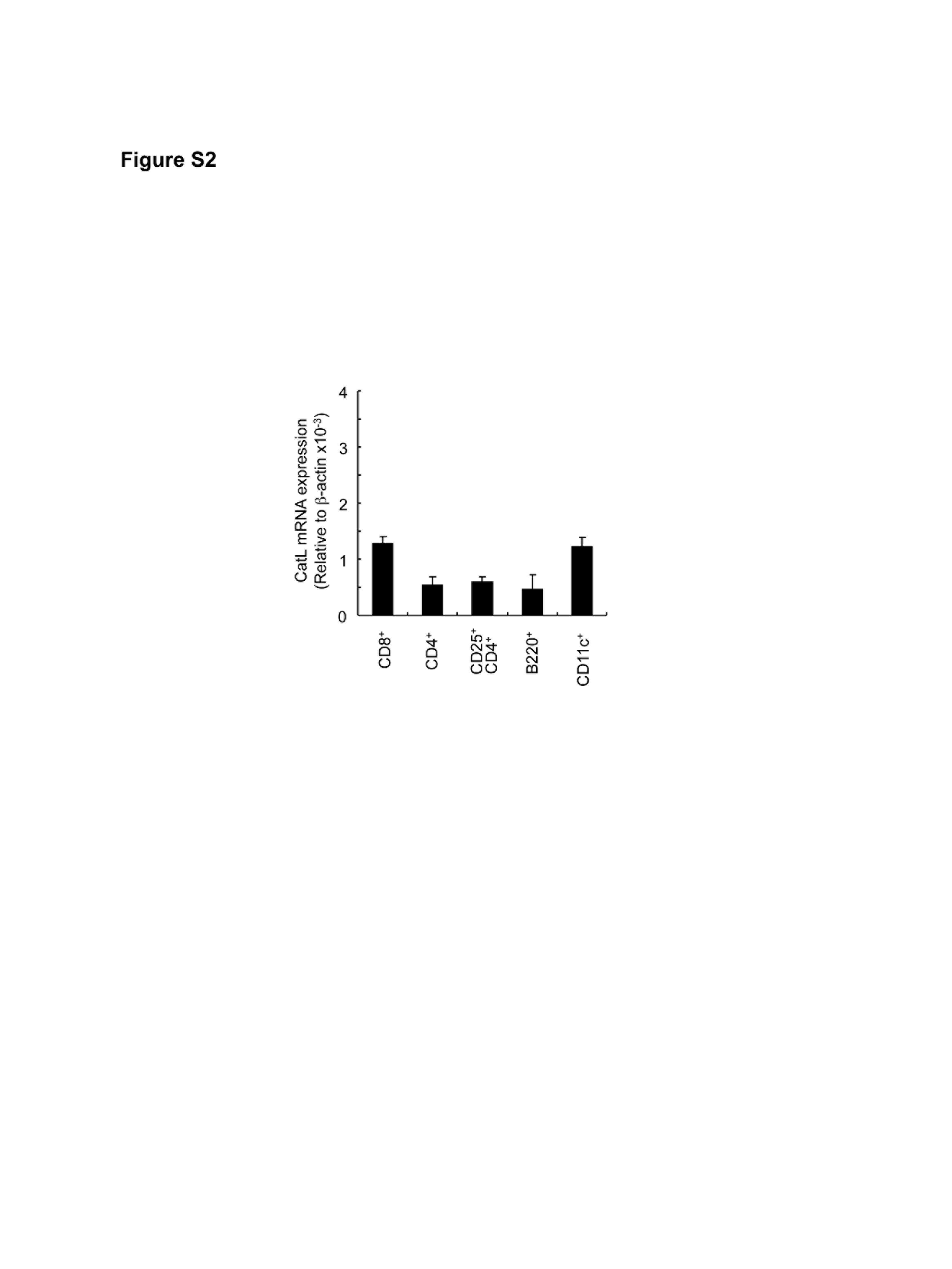

Supplement: Figure S2 — The mRNA expressions of cathepsin L in PLN cells from C57BL/6 mice. The mRNA expressions of cathepsin L in purified CD8+ T cells, CD4+ T cells, CD25+CD4+ T cells, B220+ B cells and CD11c+ dendritic cells of PLNs from C57BL/6 mice were detected by real-time PCR. Data are shown as means ± s.d. of triplicate wells, and representative of three mice. (0.20 MB TIF) [file pone.0012894.s002.tif]

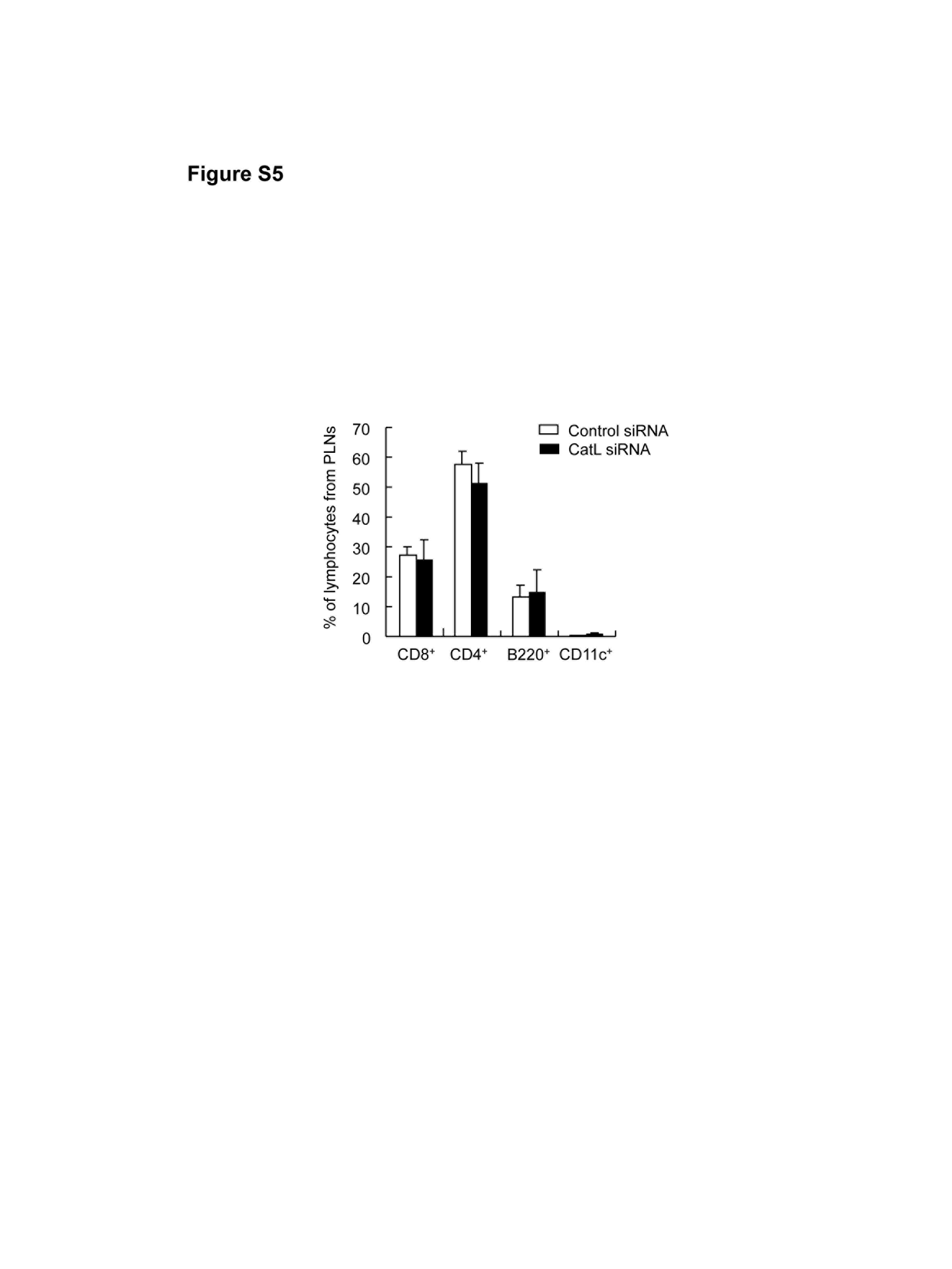

Supplement: Figure S5 — The population of PLN cells from the mice administered with control siRNA or CatL siRNA. The graph shows the mean frequency of CD8+ T cells, CD4+ T cells, B220+ B cells and CD11c+ dendritic cells. Data are shown as means ± s.d. of 3 mice. (0.21 MB TIF) [file pone.0012894.s005.tif]
